# Reaching skills in six-month-old infants at environmental and biological risk

**DOI:** 10.1371/journal.pone.0254106

**Published:** 2021-07-01

**Authors:** Liz Araújo Rohr, Thais Invenção Cabral, Murilo Mageste de Moraes, Eloisa Tudella

**Affiliations:** 1 Department of Physical Therapy, Federal University of São Carlos, São Carlos, São Paulo, Brazil; 2 School of Health and Rehabilitation Sciences, The Ohio State University, Columbus, Ohio, United States of America; The Education University of Hong Kong, HONG KONG

## Abstract

**Objectives:**

To assess kinematic parameters and proximal and distal reaching adjustments of infants at biological or environmental risk and compare with reaching performance of six-month-old full-term infants without known risk factors.

**Methods:**

This blinded cross-sectional study included 62 infants at six months of age divided into three independent groups: Group with no known risk factor (NRF), 28 full-terms with no risk factors; Low SES group (LSES):19 full-terms classified as low socioeconomic status and no biological risk; Very preterm group (VPT), 15 very preterm infants at six months corrected age and no environmental risk. Infants were placed in a reclined baby chair at 45°, and a malleable and unfamiliar object was presented to the infant at 5-second intervals to elicit reaching movements.

**Results:**

Infants from LSES presented reaching duration (p = 0.032, Cohen’s f = 0.349) and movement unit (p = 0.033, Cohen’s f = 0.351) significantly higher than VPT group. Horizontal hand orientation was moderately associated with infants at environmental risk (p = 0.031; Cramer’s V = 0.30).

**Conclusion:**

Infants of low socioeconomic status perform less functional reaching movements than very preterm infants at six months corrected age. Socioeconomic status may impact more on reaching skills than biological risk. Given the importance of reaching for infant development, low-cost public health strategies are needed to identify possible delays.

## Introduction

Reaching is defined as the ability to locate and stare at an object and stretch out one or both hands toward its direction to touch or grasp it [1,2]. This skill emerges in full-term infants approximately at 3–4 months of age [3–5], characterizing one of the first stages of voluntary motor development [6]. Reaching also allows exploration and manipulation of objects in the environment.

This ability can be assessed in detail using kinematic analysis [7] and a set of variables that provide important information regarding quality of movement, such as straightness (straightness index) and smoothness (movement unit) [8,9]. Studies involving kinematic analysis showed that upper limb movement paths are more curved during reaching acquisition, with variations in speed, movement units, and duration [3,8–12]. Efficient reaching is achieved at six-month-old [13,14], and it becomes more fluent and straight according to experience and practice. Infants first perform bimanual reaching (proximal adjustment) [15] with hands in horizontal orientation (distal adjustment) [16] and improve this skill by performing unimanual reaching with the hand open and oriented vertically [16].

Infant development occurs in a bidirectional and non-linear way, according to interactions between personal and environmental factors, body functions and structures, and activities and participation [17]. Thus, risk factors, such as biological (prematurity, neonatal hypoxemia, or low birth weight) or environmental (family factors, environmental structure, socioeconomic status [SES], or parental educational level) [18], may negatively impact reaching acquisition and engagement in activities and participation.

Infants at biological risk and late preterm infants present less functional kinematic parameters, slower movements, increased deceleration time, and non-fluid and immature reaching behavior compared with full-terms [19,20]. On the other hand, very preterm infants perform similar movement frequency and units than healthy full-term infants, indicating efficient reaching tasks [21]. However, these infants use bimanual strategies more often and present more curved reaching paths than full-term infants. Furthermore, reaching in very preterm infants is associated with development of other areas (e.g., language and cognition) and influenced by frequency of bimanual strategies, differing from the impact of kinematic parameters (e.g., straightness index and movement unit) observed in extremely preterm infants [22].

Evidence regarding reaching development in infants at environmental risk is scarce. The study conducted by Cabral (2017) [23] observed lower reaching performance (i.e., higher deceleration index) in orphaned infants than non-orphaned healthy infants. Characteristics of physical environments (i.e., home and social environments) are important for development and contribute to interaction and personal interest of the infant [24]. Clearfild et al., (2014) [25] identified delayed exploratory behavior (transfer and spin) in infants with low SES compared with infants with high SES, probably due to reduced repertoire of fine motor skills and environment with limited stimuli. Thus, environments with few affordances and opportunities for exploration may be unfavorable for developing fine motor skills, such as manual reach.

The literature regarding reaching skills in infants at risk is still scanty and inconsistent, and associations between infant reaching skills and risk factors are not clear. In this context, it is necessary to understand the development of these skills in infants exposed to different risk conditions since environmental and biological risks may interfere with skills development and maturation. Thus, this study aimed to identify differences in kinematic parameters and proximal and distal reaching adjustments in infants at biological risk (very preterm) and environmental risk (low SES) and compare reaching performance with six-month-old full-term infants without risk factors. We hypothesized that infants at biological and environmental risk would be less functional and present similar kinematic parameters than infants without risk.

## Methods

### Ethical procedures

This blinded cross-sectional study was approved by the research ethics committee of the Federal University of São Carlos (UFSCar) (Brazil) (no. 79741917.8.0000.5504), and followed the resolution 466/12 of the National Health Council and the Declaration of Helsinki. The parents/caregivers of the infants gave written informed consent.

### Participants and eligibility criteria

Full-term infants (≥ 37 gestational weeks) at six months of age and very preterm infants (28 to 32 gestational weeks) at six months corrected age of both genders were eligible for the study [26]. Corrected age was calculated by subtracting the number of weeks the infant was born before 40 weeks of gestation from the infant’s chronological age. Infants without signs of neurological impairment, congenital abnormalities in the central nervous system, musculoskeletal disorders, genetic syndromes, congenital infections, sensory deficits, and whose parents accepted to participate by signing the informed consent form were included.

Poverty income ratio (PIR) and maternal educational level determined SES [27,28]. PIR is the ratio of household income to the poverty level specific to family composition and geographic area and can be classified as high SES (maternal education more than high school and PIR ≥ 2), middle SES (high school education regardless of PIR value), and low SES (maternal education less than high school and PIR < 2) [27,28].

#### Recruitment

All infants were recruited from the maternity hospital at Santa Casa de Misericórdia in the city of São Carlos (Brazil). After identification, parents/caregivers of infants were contacted by telephone and invited to participate in the study. Three hundred forty-seven telephone calls were performed: 170 did not answer, 38 were not interested in participating, and seven did not live in the city. Of those who agreed to participate, 44 did not attend the assessment, 16 did not meet eligibility criteria and 8 were excluded due to constant crying during evaluation. Also, two infants classified as low SES were excluded from final analysis because they did not perform reaching movements during assessments. All parents/caregivers voluntarily participated in the study, and displacement was performed without costs.

The study included 62 six-month-old infants from both genders (51.61% males) divided into three independent groups: no known risk factor (NRF): 28 full-terms without known risk factors; Low SES group (LSES): 19 full-terms classified as low SES with no biological risks; Very preterm group (VPT): 15 very preterm infants at six months corrected age and without low SES Table 1.

**Table 1:** Infants’ characterization.

Variable	NRF	LSES	VPT
**Gestational age (weeks)**	39.07±1.24	39.47±1.07	29.46±0.91
**Current age (days)**	197.78±9.1	194.52±8.88	189.46±7.48
**Birth weight (kg)**	3.283±0.43	3.263±0.44	1.244±0.38
**Current weight (kg)**	7.874±1.24	7.718±1.02	7.735±1.47
Length at birth* **(cm)**	48.64±1.56	48.68±1.78	37.70±5.89
**Current length (cm)**	67.60±2.93	67.00±3.06	67.13±3.59
Apgar 1^st^ min**	8.95±0.57	8.05±1.56	6.07±1.70
Apgar 5^th^ min**	9.81±0.50	9.41±1.06	8.46±0.66
**PIR**	7.55±10.33	1.21±0.50	4.79±3.97
**Mother educational level (%)**			
Incomplete middle school	1 (3.57)	15 (71.42)	1 (6.67)
Complete middle school	0 (0)	2 (9.52)	0 (0)
Incomplete high school	6 (21.42)	4 (19.04)	3 (20)
Complete high school	11 (39.28)	0 (0)	8 (53.33)
Incomplete higher education	2 (7.14)	0(0)	1 (6.67)
Complete higher education	8 (28.57)	0(0)	2 (13.33)

NRF: Group with no known risk factor; LSES: Low socioeconomic status group; VPT: Very preterm group.

*Data missing from the birth certificate for an infant from NRF.

**Data missing from the birth certificate for seven infants from NRF, three from LSES, and two from VPT.

Kg: Kilograms.

cm: Centimeters.

min: Minute.

PIR: Poverty income ratio.

### Procedures

First, a questionnaire developed by the authors and composed of birth, personal, and sociodemographic information was applied to parents to identify environmental and biological risks and characterize infants (S1 and S2 Files). Right after, reaching movements were analyzed quantitatively and qualitatively. After the assessment, all caregivers received a booklet containing guidance on how to stimulate infant development.

#### Assessment of reaching

**Fig 1:**
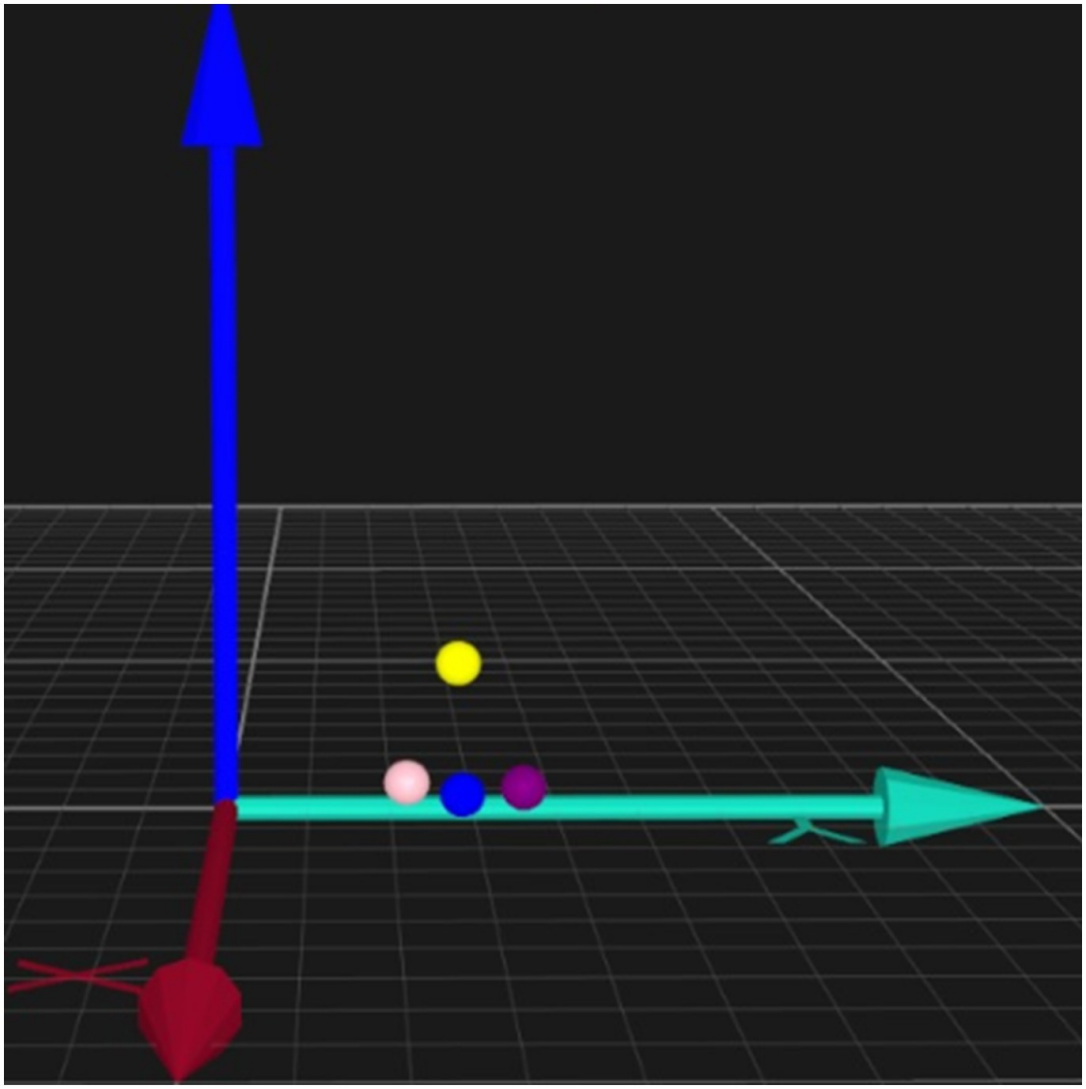
XYZ coordinates and markers. Markers: Yellow: Head; pink: Right wrist; purple: Left wrist; blue: Trunk.

**Fig 2:**
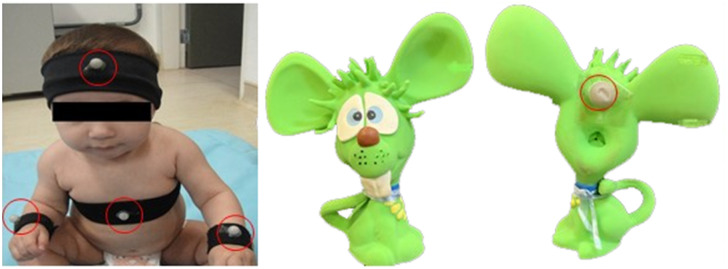
Marker’s position and malleable object.

A three-dimensional motion capture system (Qualysis, Gothenburg, Sweden) composed of five stroboscopic cameras assessed kinematic variables using a frequency of 200 Hz. X, Y, and Z coordinates were considered in the sagittal (anteroposterior), frontal (mediolateral), and longitudinal (superior-inferior) planes, respectively (Fig 1). Four spherical passive markers were positioned on the infant’s head, right and left wrists, and trunk with double-sided hypoallergenic tape. A fifth maker was placed on a malleable object unfamiliar to the infant (Fig 2).

Reaching of all infants was assessed by the same researcher, positioned in front of the infant [19,29]. Infants were placed in a baby chair reclined 45° from the horizontal, and the researcher always presented the object in the midline (shoulder height) of the infant. After reaching, the object was taken away and presented again at 5-second intervals. The researcher continued performing these movements to avoid habituation even if infants did not initiate a new reaching movement. This procedure was performed for one minute.

Only successful reaching movements were analyzed. A reach was considered successful when one or two hands touched the object, whether followed by grasping or not [1–3,30]. The beginning of a reach was defined as the first frame where the arm initiated an uninterrupted movement toward the object, deviating from middle waistline. The end of a reach was defined as the first frame the infant touched the object [3,30–33].

#### Kinematic variables

The following spatio-temporal variables were analyzed: 1) straightness index (i.e., straightness of the reach), calculated as the ratio between the shortest distance that could have been traveled to reach the object and the actual distance traveled by the hand. A value of one represented a straight approach [8,9]; 2) reaching duration, calculated as the time between the beginning and end of the movement [8,9]; 3) movement unit (i.e., the number of decelerations and accelerations used to correct the trajectory), calculated as the number of maximum velocities between two minimum velocities, where the difference exceeded one cm/s^(2)^—the lower the movement unit, the smoother the reaching movement [7]; 4) mean velocity, obtained as the ratio between hand displacement and reaching duration [8,9,29,34] 5) peak velocity, corresponding to the highest velocity achieved during the movement [2]; 6) deceleration time (time taken to decelerate the movement), calculated as the time between peak velocity and the end of the reach [8,19].

#### Qualitative analysis of reaching

Frequency of reaches was calculated as the number of successful reaching movements over one minute. Quality of reaching was assessed through proximal (uni- or bimanual) and distal adjustments when the hand of the infant touched the object.

*Proximal adjustments*. Proximal reaching adjustments indicates whether reaching was performed with one or both hands and were classified into: a) unimanual, when reach was accomplished with one hand [29,30]; b) bimanual, when reach was accomplished with both hands simultaneously toward the object and touched it, or when hands reached the object with a difference of ≤ 67 frames (frames per second) [29,30].

*Distal adjustments*. Distal adjustments refer to hand position when touching the object. The following distal adjustments were considered: a) hand orientation, corresponding to hand position when the infant touched the object and classified as: horizontal (palm faced downwards with forearm pronated), vertical (palm oriented toward the infant’s midline with forearm in neutral position), and oblique (hand in an intermediary position between horizontal and vertical positions) [16,30]; b) hand opening, referred to the position of fingers at the touching moment and classified as: open (fingers fully extended or slightly flexed), closed (fingers completely flexed), and semiopen (fingers between open and closed positions) [35]; c) hand and fingers surface area of contact, indicating whether the touch was performed with the ventral or dorsal region of the hand.

Two blinded researchers were responsible for data processing. Kinematic data processing was performed using customized Matlab^®^ 9.2 scripts, with a 4^th^-order Butterworth filter and cut-off frequency of 6Hz. Assessment of proximal and distal adjustments was recorded by a camera, and qualitative analyses were performed using Kinovea 0.8.21.

### Inter-rater reliability

Cohen’s Kappa evaluated inter-rater reliability in 20% of the infants. Inter-rater reliability was 0.74 (moderate, p<0.001) for proximal adjustments, 0.87 (strong, p<0.001) for hand orientation, 0.83 (strong, p<0.001) for hand and fingers opening, and 0.78 (moderate, p<0.001) for hand and fingers surface area of contact.

### Statistical analysis

Sample size was calculated (G*power software, v.3.1.9.2, Kiel, Germany) using mean and standard deviation of reaching frequency of a pilot study conducted with 18 participants. With an effect size of 0.52, 80% statistical power, and alpha value of 0.05, a minimum of 39 participants (i.e., 13 participants per group) was estimated.

Shapiro-Wilk and Levene’s test verified data normality and homogeneity of variance, respectively. Frequency of reaches, mean velocity, peak velocity, movement unit, and deceleration time were averaged and compared between groups using ordinary one-way analysis of variance (ANOVA), followed by Tukey’s post hoc. Straightness index was compared between groups using Kruskal-Wallis test, followed by Dunn’s post hoc.

According to data normality, effect sizes were calculated for all data using Cohen’s f or epsilon-squared (ε^2^) to avoid type II error. The former was interpreted as small (Cohen’s f = 0.10), medium (Cohen’s f = 0.25), and large (Cohen’s f = 0.40) [36], while the latter comprised coefficient values between 0 (no relationship) and 1 (perfect relationship) [37]. Relationships between kinematic variables in all groups were performed using Pearson’s correlation coefficient (r) and interpreted as weak (0.10–0.39), moderate (0.40–0.69), strong (0.70–0.89), and very strong (0.90–1.00) [37].

Associations between risk factors (environmental and biological) and proximal (unimanual and bimanual) and distal adjustments (hand orientation, hand opening, and hand and fingers surface area of contact) were verified using Chi-squared test. Cramer’s V assessed the strength of the association and was interpreted as weak (<0.299), moderate (0.300–0.499), and strong (>0.500) [38].

All statistical analyses were performed using the Statistical Package Social Science software version 22 (IBM. Corp.^®^, EUA), and the significance level was set at α = 0.05 (two-tailed).

## Results

Three hundred fifty-nine reaching movements were performed, of which 305 were considered valid for analysis. Regarding kinematic variables, four reaches were excluded during data processing; thus, 301 reaches were analyzed.

**Fig 3:**
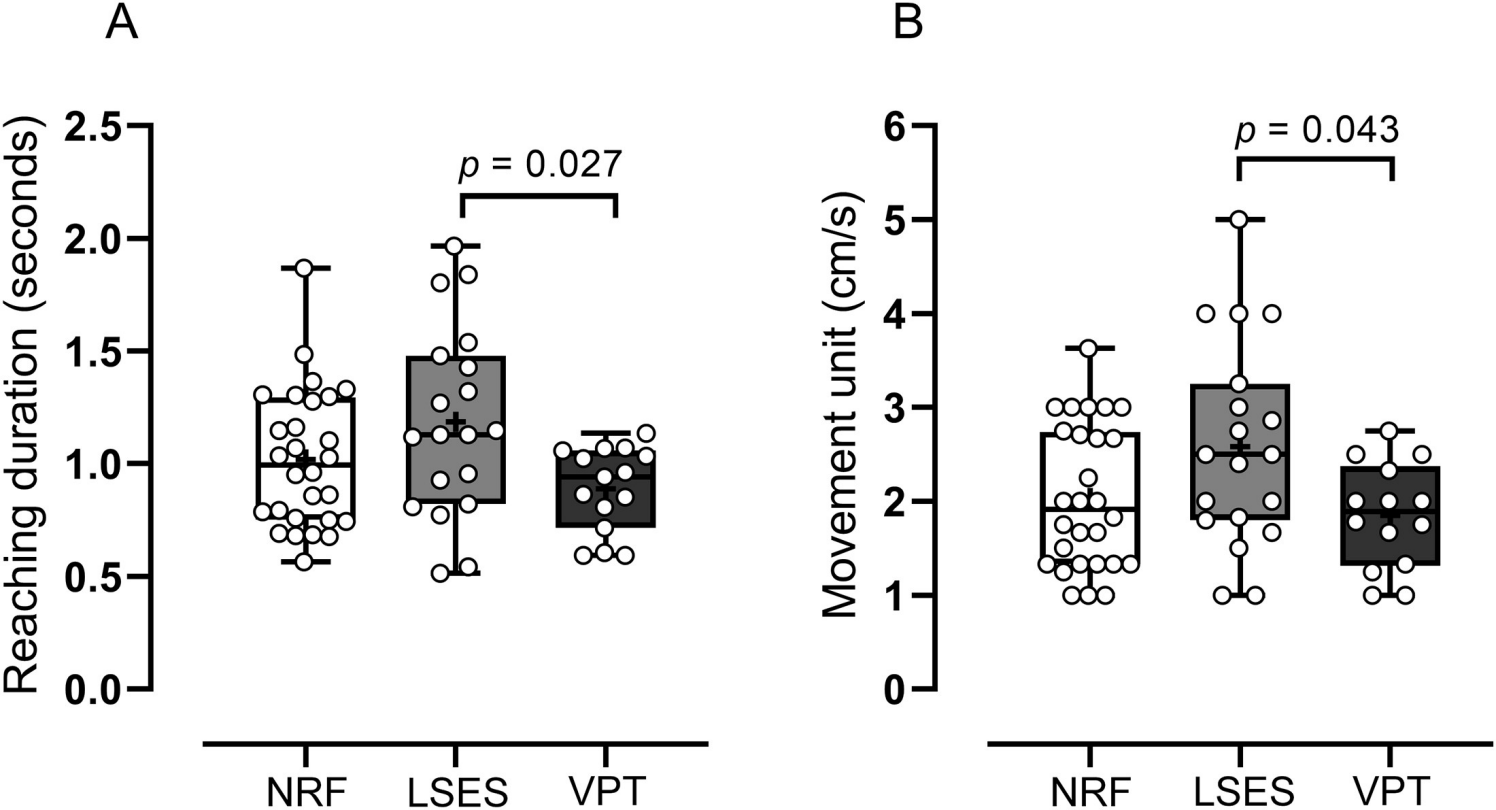
Reaching duration (A) and movement unit (B) between groups. Center lines indicate median and plus signs show mean values. Dots represent each infant. Upper and lower limits of each box represent the 75^th^ and 25^th^ percentiles, respectively. Whiskers denote minimum and maximum values. cm/s: Centimeter per second. Significance is represented by post hoc p-values.

The frequency of reach was higher in the NRF group (5.36 ± 2.36) than LSES group (4.17 ± 2.31) and VPT group (5.33 ± 2.72); however, this difference was not statistically significant (p = 0.141). Reaching duration (p = 0.032, Cohen’s f = 0.349) and movement unit (p = 0.033, Cohen’s f = 0.351) were significantly higher in LSES group than VPT group (Fig 3). Conversely, frequency of reach (p = 0.141), straightness index (0.401), mean velocity (p = 0.539), peak velocity (p = 0.266), and deceleration time (p = 280) were not different between groups Table 2.

**Table 2:** Mean, standard deviation, and interquartile range for kinematic variables in each group.

	Group									ES	p-value			
Variable	NRF			LSES			VPT							
	M (SD)	IQR 25%	IQR 75%	M (SD)	IQR 25%	IQR 75%	M (SD)	IQR 25%	IQR 75%			NRF vs LSES	NRF vs VPT	LSES vs VPT
**SI**	0.72 (0.14)	0.70	0.80	0.68 (0.14)	0.57	0.76	0.74 (0.13)	0.65	0.83	0.029^#^	0.401			
**RD (s)**	1.02 (0.31)	0.75	1.30	1.19 (0.42)	0.82	1.48	0.89 (0.19)	0.72	1.06	0.349**	0.032*	0.206	0.418	0.027*
**MU**	2.04 (0.77)	1.00	2.74	2.58 (1.09)	1.80	3.25	1.85 (0.56)	1.31	2.37	0.351**	0.033*	0.084	0.775	0.043*
**MV (cm/s)**	21.98 (8.80)	14.48	28.66	19.66 (7.17)	14.38	25.13	21.71 (6.49)	1.34	26.76	0.131**	0.539			
**PV (cm/s)**	54.43 (20.49)	43.08	67.95	48.14 (17.18)	34.92	54.19	59.50 (23.07)	38.78	79.32	0.211**	0.266			
**DT (%)**	54.51 (17.73)	44.75	68.22	48.74 (18.90)	36.78	56.98	58.86 (19.20)	44.14	76.08	0.209**	0.280			

Group description: NRF: Group with no known risk factor; LSES: Low socioeconomic status group; VPT: Very preterm group.

ES: Effect size.

M: Mean.

SD: Standard deviation.

IQR: Interquartile range.

Kinematic variables: SI: Straightness index; RD: Reach duration in seconds; MU: Movement unit; MV: Mean velocity in centimeters per second; PV: Peak velocity in centimeter per second; DT: Deceleration time in percentage.

*p<0.05.

^#^ε^2^: Epsilon-squared.

** Cohen’s f.

A weak correlation between straightness index and frequency of reach (r = 0.382; p = 0.044) and a moderate correlation between movement unit and reaching duration (r = 0.640; p = 0.0002) were found in infants from G1. Regarding infants from LSES group, only a weak correlation between movement unit and reaching duration (r = 0.813; p < 0.0001) were observed. Conversely, a moderate correlation between movement unit and reaching duration (r = 0.578, p = 0.0001) and a strong correlation between movement unit and deceleration time (r = 0.763, p = 0.0009) were identified in infants from VPT group Table 3.

**Table 3:** Correlation coefficient between kinematic variables in each group.

	Group		
Variable	NRF	LSES	VPT
SI x RD	r = -0.10; p = 0.54	r = -0.43; p = 0.06	r = -0.25; p = 0.35
SI x DT	r = 0.35; p = 0.06	r = -0.04; p = 0.85	r = -0.05; p = 0.84
SI x Freq	r = 0.38*; p = 0.04	r = -0.05; p = 0.83	r = 0.11; p = 0.67
MU x RD	r = 0.64**; p = 0.00	r = 0.81***; p<0.00	r = 0.57**; p = 0.00
MU x DT	r = 0.08; p = 0.68	r = 0.07; p = 0.76	r = 0.76***; p = 0.00
MU x Freq	r = 0.17; p = 0.38	r = -0.22; p = 0.35	r = 0.28; p = 0.29

Group description: NRF: Group with no known risk factor; LSES: Low socioeconomic status group; VPT: Very preterm group.

Variables: SI: Straightness index; RD: Reach duration in seconds; Freq: Frequency of reach MU: Movement unit; DT: Deceleration time in percentage.

Pearson correlation coefficient (r); p-value (p).

*Weak correlation.

**Moderate correlation.

***Strong correlation.

A moderate association (Cramer’s V = 0.30; p = 0.031) between hand orientation (hand in horizontal orientation) and factor Group (infants from LSES group) was also observed Table 4.

**Table 4:** Proximal and distal adjustments in each group.

	Qualitative variables										
	Freq	Proximal adjustments %		Distal adjustments %							
Group				Hand orientation			Hand and fingers opening			Hand and fingers contact surface	
		Uni	Bi	V	Ob	H	Op	SOp	C	Vent	D
**NRF**	5.36	90.62	9.38	41.72	55.05	3.23	93.11	5.99	0	97.44	0.60
**LSES**	4.17	94.44	5.56	26.55	43.40	30.05*	89.15	10.85	0	80.97	13.48
**VPT**	5.33	94.22	5.78	33.87	50.29	15.84	92.38	7.62	0	86.30	13.70

Group description: NRF: Group with no known risk factor; LSES: Low socioeconomic status group; VPT: Very preterm group.

Variables: Freq: Frequency of reach; Uni: Unimanual; Bi: Bimanual; V: Vertical; Ob: Oblique; H: horizontal; O: Open; SOp: semiopen; C: Closed; Vent: Ventral; D: Dorsal.

*Significant correlation, p<0.05.

## Discussion

This study aimed to verify differences in kinematic parameters and proximal and distal reaching adjustments between very preterm infants, infants of low SES, and infants without known risk factors. Our hypothesis that infants at biological and environmental risk would present similar kinematic parameters was not confirmed. Infants with low SES presented less efficient reaching with higher movement units and reaching duration than very preterm infants, refuting our hypothesis.

To our knowledge, this was the first study comparing reaching performance between infants at different risk conditions. Furthermore, understanding kinematic variables that best describe reaching movement in these groups and identifying what would be expected to achieve mature and efficient reaching enhances therapists’ strategies in early intervention programs.

Reaching is a fundamental skill for development. Infants explore and interact with environment through reaching movements, facilitating cognitive, perceptual, and social development [2,6,30,39]. Therefore, infants with low SES and impaired reaching may negatively impact other areas of development, resulting in limited exploration and social participation repertoires.

Data indicated that all kinematic parameters were less functional in the LSES group than NRF and VPT group. However, only movement unit and reaching duration were different between LSES group and VPT group. Compared with other infants, infants of low SES performed more sudden movements (movement unit) and spent more time (reaching duration) to complete the task, indicating less control and more time needed to finish reaching movements. Thus, we believe that these infants present a less efficient reach than very preterm infants.

Interestingly, relationships between movement unit and reaching duration were found in all groups, suggesting that these variables indicate reaching efficiency since the more fluent (movement unit) the reach, the less the time needed to perform the movement (movement duration). Relationships between straightness index and frequency of reach in infants from NRF group indicated that the straighter the movement, the fewer the corrections needed in the trajectory, favoring reaching frequency. Kinematic analysis of reaching movements is complex, and its analysis provides a better understanding of movement efficiency.

Regarding proximal and distal adjustments, only hand orientation (horizontal) associated significantly with infants from LSES group. This result suggests that infants of low SES are more prone to perform reaching movements with hands in the horizontal position, considered an immature adjustment [16,30]. This also corroborates with kinematic findings, indicating that strategies adopted to perform reaching movements are less functional and may negatively impact grasping and object manipulation.

Our results contrast those from Greco (2020) [40], who applied reaching-specific training or social training on the emergence of reaching in infants of low SES. Infants were evaluated after five days and when completed six months of age. The author observed improvements in kinematic variables and proximal and distal adjustments, regardless of training type, suggesting that infants of low SES present adequate functional reaching at six months of age.

The environment seems to play an important role in the development of reaching skills. We believe that context in which infants of low SES live may hinder opportunities to practice reaching skills and manipulate different objects and toys. This would explain why their strategies were less functional. Previous studies have shown that environments with adequate stimuli contribute positively to motor performance [41,42]. Brain growth and development are also influenced by environmental factors [43] since worse socioeconomic status may impact brain activity of infants, impairing language, attention [44], hippocampus, and amygdala development [45].

Our results differ from literature regarding preterm infants. At six and seven months corrected age, reaching movements of preterm infants are immature, curved, and slower than full-term infants [19,20]. Although bimanual reaching, curved trajectories, and compensatory movements are more frequent at eight months corrected age, frequency of reaching and movement units are similar to full-term infants [21].

Kinematic parameters of very preterm infants were not different from NRF infants. Reaching was considered straight, smooth, with movement speed and duration similar to infants in the NRF. We believe the results differed from literature because participants in our study did not present associated risk factors (i.e., the same infant was not classified as preterm and low SES). Conversely, this information is unclear in previous studies [19–21]. In our study, the researcher presented the object in the infant’s midline, differing from Grönqvist et al. (2011) [21], who probably influenced task demand using moving toys to elicit reaching movements. Also, at six months corrected age, very preterm infants presented appropriate biological factors (i.e., weight and length). Thus, the lack of environmental risk (i.e., low SES and maternal educational level) may have helped proper reaching development. Furthermore, infants were assessed at six months corrected age, indicating temporal advantage over other infants due to greater extra-uterine opportunities to practice the skill [46].

Lastly, no significant differences were found between infants at risk and without risk. We investigated reaching performance in infants with specific risk factors, such as SES and prematurity, and SES classification considered important environmental factors (maternal education, income, and number of people living in the house). Although infants did not present associated risk factors (i.e., low SES and very preterm birth), we did not use any instrument (e.g., Affordances in the Home Environment for Motor Development—Infant Scale) [47] to assess quantity and quality of stimuli received in the family environment. Affordances provided by the environment may play an important role in motor and cognitive development [48]. Thus, further studies are needed to investigate the influence of quality of affordances on development of reaching of infants with and without risks and explore relationships between development of reaching and other fine motor skills, such as manual exploration.

## Limitations

This study has limitations that should be considered. First, monitoring of reach skills was not conducted since its emergence; therefore, it is unknown whether infants of low SES presented delayed skill acquisition. Second, although none of the included infants attended rehabilitation programs, results may have been influenced by caregivers’ awareness regarding special needs, leading to information search and assistance in specialized healthcare centers. Third, our study did not include infants with more than one risk factor or evaluated house stimuli and environmental variables. Thus, future studies should emphasize premature infants with low SES and verify the role of environmental variables in early motor development since low SES may be associated with an unfavorable amount of environmental stimuli.

## Clinical implications

Our findings highlight important evidence about reaching performance in six-month-old infants at biological and environmental risk. SES seems to impact skill performance more than biological risk when these infants present no risk factors associated. The results of this study do not minimize findings regarding infants at biological risk [33,49–52]; however, we emphasize the importance of low-cost strategies (i.e., stimulation strategies for families and educators) within the scope of public health. A closer approach to the development of infants of low SES may reduce its impacts on future life. According to Bornstein et al. [53], infants at five months of age with advanced motor-exploratory competence achieve higher cognitive development at four and 10 years and higher academic levels at 10 and 14 years.

## Conclusions

At six months of age, infants of low SES perform reaching movements with less smooth trajectories than very preterm infants at six months corrected age. Also, infants of low SES are more prone to reach objects with hands in horizontal position, which is not functional for the age. Therefore, greater attention is needed for this group, given the importance of reaching for infant motor development and the lack of early intervention programs. Low-cost strategies to encourage development of reaching skills and minimize negative functional impacts of environmental factors must be investigated in future studies.

## Supporting information

S1 File Sociodemographic questionnaire. English version.
(DOCX)

S2 File Sociodemographic questionnaire. Portuguese version.
(DOCX)
