# Supplementary material for: Reaching skills in six-month-old infants at environmental and biological risk
Source: PLoS One. 2021 Jul 1;16(7):e0254106. doi: 10.1371/journal.pone.0254106 (PMC8248732; doi:10.1371/journal.pone.0254106)
Supplement: S1 File — English version. (DOCX) [file pone.0254106.s001.docx]

**Questionary**

1. **Personal information:**

Infant’s name: .......................................................................... Sex: ( ) M ( ) F

Age: .................................. Birth date: ......../......../......

Gestational age....................

Address: ...................................................................................................

Contact number: ...........................................

Mother’s name: ............................................................................................

Age: .................................. Birth date: ......../......../......

Maternal educational level: .............................. Occupation: ...............................

Family income: ..............................................

Number of people living in the house:.......................

1. **Gestational information:**

Number of pregnancies: ................................

Time between pregnancies: ..................................................................

Mother's disease: ( ) No ( ) Yes

( ) Anemia ( ) Syphilis ( ) () Diabete ( ) Toxoplasmosis ( ) Fever ( ) Rubella

( ) others: .................

Abnormalities in pregnancy: ( ) No ( ) Yes

( )Bleeding ( )Hypertension ( ) Hypotension ( ) Edema ( ) Others : ................................................................

Ingestion of toxics: ( ) No ( ) Yes

( ) Smoking ( ) Alcoholism

( ) Others: .................................

Medication Intake: ( ) No ( ) Yes

( ) Tranquilizers ( ) Vitamins ( ) Others : ......................

Rx Exposure: ( ) No ( ) Yes

Month of gestation: ...................................................

Malnutrition and/or ill-treatment: ( ) No ( ) Yes

Gestation quarter: ...................................................

**3) Birth information:**

Type of delivery:() Spontaneous ( ) Induced ( ) Forceps ( ) Caesarean Near duration: .........................

Umbilical Cord:( ) Normal ( ) Circular ( ) Knot

Some intercorrence: ...............................................................................

**4) Postnatal Information:**

Birth weight: ...............................

Stature:............... cm

CP:...................... Cm

Apgar: 1st min: ....... 5th min: ................

Crying at birth: ( ) Normal ( ) Weak ( ) High with agitation

Jaundice: duration: ...................... Days

Diseases: ( ) Erythroblastosis ( ) Convulsions ( ) Heart diseases ( ) others : ..............................

drugs:................................................................................................
