# Supplementary material for: Reaching skills in six-month-old infants at environmental and biological risk
Source: PLoS One. 2021 Jul 1;16(7):e0254106. doi: 10.1371/journal.pone.0254106 (PMC8248732; doi:10.1371/journal.pone.0254106)
Supplement: S2 File — Portuguese version. (DOCX) [file pone.0254106.s002.docx]

**Questionário**

1. **Informações pessoais:**

Nome do lactente: .......................................................................... Sexo: ( ) M ( ) F

Idade: .................................. Data de nascimento: ......../......../......

Idade gestacional:…….....................................................................................

Endereço:................................................................................................... Bairro: .................................................. Telefone: ...........................................

Nome da mãe: ............................................................................................

Idade: ..................................... Data de nascimento: ......../........./..............

Grau de escolaridade: .............................. Profissão: ...............................

Renda mensal: ..............................................

N° de pessoas que moram na residência:.......................

1. **Informações gestacional:**

N° de gestações: ................................

Quanto tempo entre as gestações: ..................................................

Doenças da mãe: (  ) Não ( ) Sim

( ) Anemia ( ) Sífilis ( ) Diabete (  ) Toxoplasmose ( ) Febre ( ) Rubéola

( ) outras: .................

Anormalidades na gravidez: (  ) Não ( ) Sim

( )Hemorragias ( )Hipertensão ( ) Hipotensão ( ) Edema (  ) Outras: ................................................

Ingestão de tóxicos: (  ) Não ( ) Sim

( ) Fumo ( ) Alcoolismo ( ) Outros: .................................

Ingestão de Medicamentos: (  ) Não ( ) Sim

( )Tranquilizantes ( ) Vitaminas ( ) Outros: ......................

Exposição ao RX: (  ) Não ( ) Sim

Mês de gestação: ...................................................

Desnutrição e/ou maus tratos: (  ) Não ( ) Sim

Época gestação: ...................................................

**3) Informações do nascimento:**

Tipo de parto:( ) Espontâneo ( ) Induzido ( ) Fórceps ( ) Cesária Duração do perto: ...............................................................

Cordão Umbilical:( ) Normal ( ) Circular ( ) Nó

Alguma intercorrência: ...............................................................................

4) Informações Pós-natal :

Peso nascimento: ...............................

Estatura: ............... cm

PC: ...................... cm

Apgar: 1º min: ……. 5º min: ................

Choro ao nascimento: ( ) Normal ( ) Fraco ( ) Alto com agitação

Icterícia: duração: ...................... dias

Doenças: ( ) Eritroblastose ( ) Convulsões ( ) Cardiopatias ( ) outras: ..............................................................................................................

Medicamentos: ................................................................................................
